# Progression-free survival versus post-progression survival and overall survival in WHO grade 2 gliomas

**DOI:** 10.2340/1651-226X.2024.40845

**Published:** 2024-10-20

**Authors:** Lisa Millgård Sagberg, Øyvind Salvesen, Asgeir Store Jakola, Erik Thurin, Eddie de Dios, Noah L.A. Nawabi, John L. Kilgallon, Joshua D. Bernstock, Vasileios K. Kavouridis, Timothy R. Smith, Ole Solheim

**Affiliations:** a Department of Public Health and Nursing, Norwegian University of Science and Technology, Trondheim, Norway; b Department of Neurosurgery, St Olavs Hospital, Trondheim University Hospital, Trondheim, Norway; c Clinical Research Unit, Department of Clinical and Molecular Medicine, Norwegian University of Science and Technology, Trondheim, Norway; d Department of Neurosurgery, Sahlgrenska University Hospital, Gothenburg, Sweden; e Institute of Neuroscience and Physiology, Department of Clinical Neuroscience, Sahlgrenska Academy, Gothenburg, Sweden; f Department of Radiology, Sahlgrenska University Hospital, Gothenburg, Sweden; g Department of Neurosurgery, Brigham and Women’s Hospital, Harvard Medical School, Boston, MA, USA; h David H. Koch Institute for Integrative Cancer Research, Massachusetts Institute of Technology, Cambridge, MA, USA; i Department of Neuromedicine and Movement Science, Norwegian University of Science and Technology, Trondheim, Norway

**Keywords:** Brain neoplasms, surrogate endpoints, response assessment criteria, prognostic factors, oncology

## Abstract

**Background and purpose:**

Progression-free survival (PFS) remains to be validated as an outcome measure for diffuse WHO grade 2 gliomas, and knowledge about the relationships between PFS, post-progression survival (PPS), and overall survival (OS) in this subset of tumors is limited. We sought to assess correlations between PFS and OS, and identify factors associated with PFS, PPS, and OS in patients treated for diffuse supratentorial WHO grade 2 gliomas.

**Material and methods:**

We included 319 patients from three independent observational cohorts. The correlation between PFS and OS was analyzed using independent exponential distributions for PFS and time from progression to death. Cox proportional hazards models were used to determine the effects of covariates on PFS, PPS, and OS.

**Results:**

The overall correlation between PFS and OS was r_s_0.31. The correlation was r_s_ 0.37 for astrocytomas and r_s_ 0.19 for oligodendrogliomas. Longer PFS did not predict longer PPS. Patients with astrocytomas had shorter PFS, PPS, and OS. Larger preoperative tumor volume was a risk factor for shorter PFS, while older age was a risk factor for shorter PPS and OS. Patients who received early radio- and chemotherapy had longer PFS, but shorter PPS and OS.

**Interpretation:**

We found a weak correlation between PFS and OS in WHO grade 2 gliomas, with the weakest correlation observed in oligodendrogliomas. Our analyses did not demonstrate any association between PFS and PPS. Critically, predictors of PFS are not necessarily predictors of OS. There is a need for validation of PFS as an endpoint in diffuse WHO grade 2 gliomas.

## Introduction

Median overall survival (OS) in adults with diffuse WHO grade 2 gliomas is approximately 15 years [1], although the range can vary considerably based on a myriad of prognostic and treatment-related factors [2]. Given this extended and relatively unpredictable survival and disease trajectory, most patients undergo multiple interventions of which the majority are triggered by radiological tumor progression. Thus, progression-free survival (PFS) represents an attractive surrogate endpoint with potential clinical utility in this population as it requires shorter follow-up time(s) and is not affected by post-progression therapies. Recently, Mellinghoff et al. demonstrated that vorasidenib increased PFS from 11.1 to 27.7 months [3], and this dual inhibitor of mutant isocitrate dehydrogenase (IDH1/2) recently received the US Food and Drug Administration (FDA) approval based on the demonstrated effect on PFS.

For PFS to be a valid surrogate endpoint that carries meaningful weight and consideration in clinical decision making, it must be shown to be clinically meaningful. For instance, treatment effects on PFS should ideally be predictive of effects on OS [4]. However, PFS is not yet a validated outcome measure in WHO grade 2 gliomas, and there is limited data available regarding the extent to which this endpoint reflects post-progression survival (PPS) and/or OS. Indeed, strong correlations between PFS and OS have been found in tumor types with more aggressive disease trajectories and shorter PPS [5], such as in high-grade gliomas [6–8]. The validity of PFS may also be treatment/intervention specific, as improvements in PFS do not always correlate with improvements in OS as is evident in ‘early’ radiotherapy for low-grade gliomas [9]. Given that many patients with WHO grade 2 gliomas will spend the majority of their disease course in the post-progression phase, knowledge about the prognostic value of PFS, and its relationship with PPS and OS, is of major clinical interest.

Using data derived from three merged observational cohorts of patients treated for diffuse supratentorial WHO grade 2 gliomas, we sought to assess correlations between PFS and OS, and to identify factors associated with PFS, PPS, and OS.

## Material and methods

### Study design and study population

We conducted a retrospective multicenter study using data from consecutively enrolled patients from three different observational cohorts: (A) St. Olavs hospital, Trondheim, Norway from 2005 to 2018, (B) Sahlgrenska University Hospital, Gothenburg, Sweden from 2007 to 2018, and (C) Brigham and Women’s and Massachusetts General Hospitals, Boston, Massachusetts, USA from 2004 to 2018. All included patients underwent primary surgery (either biopsy or resection) for a histopathologically confirmed, previously untreated IDH-mutant WHO grade 2 glioma. Other inclusion criteria were age ≥ 18 years, and follow-up ≥ 3 years (unless deceased prior to this mark). Exclusion criteria included lack of postoperative magnetic resonance images (MRIs) taken <4 months after surgical resections (i.e., lack of baseline status) and infratentorial tumor location(s).

### Endpoints

PFS was defined as days from time of diagnosis (i.e., initial biopsy or resection) to first tumor progression or to death from any cause; PPS was defined as days from PFS to OS; OS was defined as days from time of diagnosis to death from any cause [10]. The Response Assessment in Neuro-Oncology (RANO) criteria were used to evaluate tumor progression [11]. Cases where malignant transformation was initially suspected on contrast-enhanced T1-weighed MRI sequences but later MRIs and the clinical course indicated that it was radiation necrosis, were not recorded as progressive disease. RANO-assessments were conducted separately at each center, and the total number of MRI scans available for assessment until progression or end of follow-up was recorded.

### Other variables

Baseline clinical characteristics, radiological data, and treatment data were retrieved from electronic medical records and imaging systems at each center. Volumetric assessments of preoperative and postoperative tumor volumes were done using the 3D Slicer software (http://www.slicer.org). For patients who underwent a biopsy as primary surgery followed by resection within 90 days, postoperative volumes were assessed after the resection. Histopathology was reclassified according to the latest updated WHO-classification from 2021 [12]. In sum, IDH-mutant tumors with 1p19q co-deletion were classified as oligodendrogliomas and tumors without 1p19q co-deletion were classified as astrocytomas. Cases with missing 1p19q status were classified as unspecified gliomas.

### Statistical analyses

Statistical analyses were performed using IBM SPSS Statistics for Windows, version 29.0.1 (Armonk, New York: IBM Corp) and R version 4.1.2 (R Foundation for Statistical Computing, Vienna, Austria). Descriptive statistics are reported as medians and ranges (skewed continuous data) or as frequencies and percentages (categorical data). To identify high-risk tumors, patients were categorized into risk groups based on preoperative tumor volume (cut off 43.1 mL), postoperative residual tumor volume (cut off 4.6 mL), and chemotherapy treatment (yes/no), as described by Hervey-Jumper et al. [2] The Spearman correlation coefficient between PFS and OS was estimated using independent exponential distributions for PFS and time from progression to death. Cox proportional hazards models were used to determine the effect of covariates on PFS, PPS, and OS. Patients were censored on the last day they were known to be alive and free of tumor progression. The proportional hazards assumption was assessed using the Schoenfeld residual test. Multicollinearity was assessed from the covariance matrix of parameter estimates and Pearson correlations between pairs of covariates. Statistical significance was set to *p* ≤ 0.05.

## Results

A total of 319 patients with supratentorial WHO grade 2 gliomas were included in the study. Patient and treatment characteristics are presented in Table 1. Further treatment details are presented in Supplementary Table 1 and 2.

**Table 1:** Patient and treatment characteristics.

Variable	All *N* = 319	Hospital A *N* = 65	Hospital B *N* = 70	Hospital C *N* = 184
**Age, median (range)**	39 (18–82)	39 (18–69)	45.5 (18–82)	36 (18–79)
**Sex, No. (%)**				
Female	138 (43)	22 (34)	31 (44)	85 (46)
Male	181 (57)	43 (66)	39 (56)	99 (54)
**Type of surgery, No. (%)**				
Resection	301 (94)	62 (95)	66 (94)	173 (94)
Biopsy only	18 (6)	3 (5)	4 (6)	11 (6)
**Histopathology, No. (%)**				
Astrocytoma	138 (43)	32 (49)	35 (50)	71 (39)
Oligodendroglioma	172 (54)	33 (51)	35 (50)	104 (57)
Unspecified	9 (3)	0 (0)	0 (0)	9 (5)
**Preoperative tumor volume in mL, median (range)**	32.2^a^ (0.8 – 227)	32.1 (0.8 – 179.4)	47.1 (1.8 – 227)	29.9^a^ (1.6 – 194.1)
**Postoperative residual tumor volume ≤ 4.6 mL, No. (%)**	159 (50)	37 (57)	29 (41)	93 (51)
**Radio- and chemotherapy within 6 months, No. (%)**	57 (18)	9 (14)	18^b^ (26)	30 (16)
**Risk groups** ^**c**^ **, No. (%)**				
1 (worst)	84 (26)	17 (26)	25 (36)	42 (23)
2 (intermediate)	129 (40)	27 (42)	29 (41)	73 (40)
3 (best)	97 (30)	21 (32)	16 (23)	60 (33)

a One patient registered with 0 mL, and therefore excluded from analysis (assumed to be a plotting error).

b Missing data in one patient.

c According to Hervey-Jumper et al. Patients with unspecified histopathology excluded.

The median follow-up time from diagnosis to either death or the end of follow-up was 7.4 years (range 0.9 – 17.8). The number of MRI-scans available for assessment was registered in patients until progression (median 9, range 2 – 50), or until end of follow-up in patients without progression (median 16.5, range 5–81).

In total, 235 of 319 patients progressed according to the RANO criteria during follow-up (74%), and median PFS was 4.4 years (95% confidence interval [CI]: 3.8, 5.0). Median OS was not reached, but 53 of 319 patients died during the follow-up period (17%). Of these, three (6%) died from causes other than glioma. The calculated overall correlation between PFS and OS was *r_s_* = 0.31 (95% CI: 0.24, 0.38). The correlation between PFS and OS was stronger in astrocytoma (*r_s_* = 0.37, 95% CI: 0.28, 0.47) than in oligodendroglioma (*r_s_* = 0.19, 95% CI: 0.10, 0.29). Sensitivity analyses in the patients who did not undergo early radio- and chemotherapy (i.e., within 6 months after first surgery) revealed similar results (*r_s_* = 0.25, 95% CI: 0.18, 0.32). In this group, 37/262 patients died during follow-up (14%). There was a stronger correlation in the patients who received early adjuvant treatment (*r_s_* = 0.73, 95% CI: 0.52, 0.86) where 16/57 patients (28%) died during follow up.

The potential impact of established prognostic factors on PFS, PPS, and OS were analyzed using Cox proportional hazards models (Table 2). The multicollinearity in the models was considered acceptable, and the strongest correlation was between preoperative tumor volume and postoperative residual tumor volume (*r* = 0.68). As seen from the multivariable analyses, patients with astrocytoma (*p* = 0.011), and larger preoperative tumor volumes (*p* = 0.002) had shorter PFS while patients who had undergone early radio- and chemotherapy (*p* = 0.026) had a longer PFS. Age at diagnosis and postoperative tumor volumes were not associated with PFS, but larger postoperative volume was associated with shorter PFS (Hazard Ratio (HR): 1.009; *p* = 0.046) in a multivariable post hoc subgroup analysis in astrocytomas. As also presented in the table, patients with astrocytoma (*p* < 0.001), patients who received early radio- and chemotherapy (*p* < 0.001), and patients who were older at diagnosis (*p* = 0.001) had shorter PPS. Neither PFS, pre-, nor postoperative tumor volumes were associated with PPS, although patients with larger residual tumors had shorter PPS (HR: 1.016; *p* = 0.014) in a multivariable post hoc subgroup analysis in astrocytomas. Furthermore, patients with astrocytoma (*p* < 0.001), patients who received early radio- and chemotherapy (*p* = 0.003), and patients who were older at diagnosis (*p* = 0.002) had shorter OS. Pre- and postoperative tumor volumes were not statistically significant independent predictors for OS, but patients with larger residual tumor volumes had shorter OS (HR: 1.019; *p* < 0.001) in a multivariable post hoc subgroup analysis of astrocytomas.

**Table 2:** Predictors of progression-free survival, post-progression survival, and overall survival.

Variable	Progression-free survival (PFS)						Post-progression survival (PPS)						Overall survival (OS)					
	Univariable analyses			Multivariable analyses			Univariable analyses			Multivariable analyses			Univariable analyses			Multivariable analyses		
	HR	95% CI	*p*	HR	95% CI	*p*	HR	95% CI	*p*	HR	95% CI	*p*	HR	95% CI	*p*	HR	95% CI	*p*
Astrocytoma histopathology (y/n)	1.406	1.085, 1.822	**0.010**	1.409	1.081, 1.837	**0.011**	2.889	1.569, 5.322	**<0.001**	3.282	1.719, 6.264	**<0.001**	3.557	1.931, 6.552	**<0.001**	4.477	2.367, 8.469	**<0.001**
Age at diagnosis, years (continuous)	0.993	0.982, 1.003	0.189	0.995	0.984, 1.006	0.341	1.039	1.016, 1.061	**<0.001**	1.041	1.016, 1.067	**0.001**	1.031	1.009, 1.054	**.0005**	1.037	1.013, 1.061	**0.002**
Preoperative tumor volume, mL (continuous)	1.004	1.001, 1.007	**0.005**	1.006	1.002, 1.097	**0.002**	1.010	1.005, 1.015	**<0.001**	1.004	0.996, 1.012	0.360	1.012	1.007, 1.017	**<0.001**	1.006	0.999, 1.013	0.111
Postoperative residual tumor volume, mL (continuous)	1.002	0.997, 1.007	0.401	0.997	0.991, 1.004	0.392	1.014	1.007, 1.022	**<0.001**	1.010	0.998, 1.021	0.096	1.015	1.008, 1.022	**<0.001**	1.010	0.999, 1.021	0.065
Radio- and chemotherapy < 6 months (y/n)^a^	0.688	0.468, 1.012	0.058	0.632	0.422, 0.946	**0.026**	5.248	2.889, 9.532	**<0.001**	3.422	1.769, 6.621	**<0.001**	3.561	1.934, 6.558	**<0.001**	2.583	1.370, 4.871	**0.003**
PFS, days (continuous)							1.000	0.999, 1.000	0.190	1.000	1.000, 1.000	0.590						

HR: Hazard Ratio; CI: confidence interval.

a Time-dependent variable.

Bold values = p ≤ 0.05.

**Figure 1:**
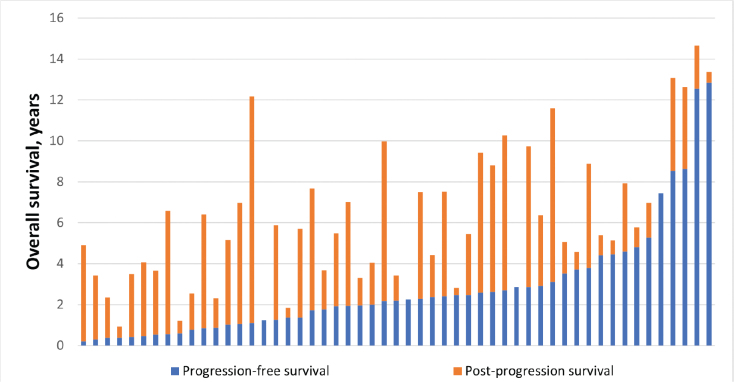
The relationship between progression-free survival and overall survival. Each patient is represented by a horizontal bar. Patients are ranked in order of their progression-free survival in years.

The relationship between PFS, PPS, and OS in patients with diffuse supratentorial WHO grade 2 gliomas who met both endpoints is illustrated in Figure 1. Of note, OS is not accurately reflected by their PFS.

## Discussion

In this multicenter observational study, we found only a weak correlation between PFS and OS in patients with diffuse supratentorial IDH-mutant WHO grade 2 gliomas, with the weakest correlation observed in those with oligodendroglioma. Furthermore, PFS did not appear to be associated with PPS. This indicates that the duration of the post-progression phase may be independent of the often-shorter pre-progression phase, perhaps due to the impact of subsequent interventions. While an astrocytoma diagnosis was an independent risk factor for shorter PFS, PPS, and OS, the influence of other established prognostic factors on these endpoints were inconsistent. Larger preoperative tumor volume was a strong independent predictor for shorter PFS but not for PPS and OS, and older patients had shorter PPS and OS, but not PFS. Furthermore, patients who received early radio- and chemotherapy had longer PFS, but shorter PPS and OS. As such, while PFS may appear to be an attractive surrogate endpoint in studies of diffuse WHO grade 2 gliomas, we must emphasize that better PFS may not necessarily result in better PPS or OS. However, given the enhanced life expectancy of patients with molecularly-defined WHO grade 2 gliomas with modern treatments, randomized data centered solely on OS could potentially delay therapeutic progress (e.g., the case for vorasidenib) [3]. Nevertheless, validation of PFS as a surrogate endpoint in diffuse WHO grade 2 gliomas is still warranted.

Since the degree of correlation between PFS and OS is affected by potential second-line treatment(s) and the length of the post-progression phase [5], the weak correlation between these variables in our observational data was not unexpected. In patients with WHO grade 2 gliomas the median OS is 15 years [13], and recurrence/progression is often treated with repeated surgery and/or radio- and chemotherapy. Thus, PPS is often very long, especially in younger patients with oligodendrogliomas. Accordingly, we found the correlation between PFS and OS to be particularly strong in a subgroup of patients with comparatively poor prognoses, namely those selected to receive early radio- and chemotherapy. Still, for the many patients with diffuse WHO grade 2 glioma who experience progression relatively early, it may be reassuring to know that their preceding PFS is not necessarily predictive of their PPS. Critically, the potential validity of PFS as an outcome measure may also be intervention-specific; for example, strong effects of bevacizumab on PFS resulted in FDA approval for glioblastoma [14], while follow-up studies showed no effect on OS [15, 16]. For diffuse low-grade gliomas diagnosed from 1986 to 1997, early radiotherapy alone was shown to improve PFS, but failed to improve OS [9].

We found older age at diagnosis to be a predictor for shorter PPS and OS, but not for PFS. A few patients also died from causes other than glioma, and it is perhaps no surprise that the importance of age with respect to predictive value increases with time over what is an often a long disease trajectory. Larger preoperative tumor volume was also associated with shorter PFS, but not with PPS or OS. However, progression (as defined by RANO) of a small tumor might have less impact on OS and be biologically and prognostically different than a similar progression of a large tumor. Measuring radiological progression may also potentially be easier or more accurate in larger lesions. Interestingly, we failed to identify a statistical association between postoperative tumor volumes and OS, although surgical resection is a well-known prognostic factor [17]. This may be due to the relatively small number of deaths in our cohort. However, as pre- and postoperative volume were correlated in our data, we cannot clearly identify which of the two is more important in the PPS and OS analyses. Nevertheless, in a post hoc subgroup analysis in astrocytomas, larger postoperative volume was associated with both shorter PFS, PPS, and OS.

The only consistent and strong predictor across models for PFS, PPS, and OS in this study was histopathology. Astrocytoma is a well-known prognostic factor for both PFS and OS [2], as astrocytomas grow much faster than oligodendrogliomas [18, 19]. The responses to both surgery [2, 13, 20] and adjuvant therapy [21] are also different between these tumors, and although usually assessed together, diffuse WHO grade 2 astrocytoma and WHO grade 2 oligodendroglioma arguably represent two almost different diseases. In observational data like this, more aggressive treatment may also be offered to patients with a higher perceived risk for malignant progression or an aggressive disease course. This likely explains the shorter PPS and OS in patients receiving early radio- and chemotherapy in our study, which aligns with the findings of a recent large predictor study [2], contrasting the evidence from the RTOG 9802 trial that demonstrated a large OS benefit of radiotherapy followed by adjuvant procarbazine, CCNU, and vincristine (PCV) in perceived high-risk patients [22]. The observed longer PFS after early radio- and chemotherapy may reflect a true treatment effect [9], but could also possibly be explained by a more difficult PFS assessment after radiotherapy due to treatment-induced image changes.

Whether PFS is clinically meaningful and associated with functional status, symptoms, or health-related quality of life (HRQoL) in diffuse WHO grade 2 gliomas is also still unproven. By postponing tumor progression, symptoms of progressive disease and side effects of further treatments are hopefully avoided. This framework has been previously utilized as hypotheses for numerous studies and was also the rationale behind the INDIGO trial [3]. Although all of these tumors will grow [23], patients with more stable disease are also spared the presumed psychological burden of having a progressive disease. An association between tumor progression and worse HRQoL has been demonstrated on numerous occasions in patients with high-grade gliomas [24–27] and in high-risk low-grade gliomas treated with radiotherapy between 2005 and 2010 [28]. However, as also acknowledged in the RANO criteria, radiological progression may be seen in clinically stable patients and clinical deterioration may occur without radiological progression. Also in the present study, many patients received second-line treatments or reoperations at a time where they were radiologically stable according to the RANO criteria. In a recent study based on pooled trial data on 5539 patients with grade 2–4 glioma, only about half of the patients maintained their HRQoL in the PFS period [29]. A systematic review of seizure outcomes after treatment in low-grade gliomas also found discrepancies between MRI findings and seizure activity [30]. Consequently, it could be possible that clinical outcome assessments have greater clinical importance or prognostic value than PFS in WHO grade 2 gliomas. This has also been described by the RANO group [11], who have proposed guidelines for using seizure control to assess the efficacy of treatment [31].

For PFS to be a valid surrogate endpoint, reliable assessments of both radiological and clinical disease progression are needed. In diffuse WHO grade 2 gliomas, MRI assessments have high interobserver variability due to the bidimensional measurements, which are part of RANO criteria [32]. Most tumors do not exhibit contrast enhancement and radiological progression is often determined based on small, incremental changes on two-dimensional T2/FLAIR images [33]. According to RANO-criteria, the progression line-in-the-sand of the ever growing gliomas is arbitrarily set at 25% increase in the sum of products of perpendicular diameters in oddly shaped tumors in T2/FLAIR images. Still, from a clinical perspective, there is no difference between a tumor that grows by 24% versus 25%. In diffusely infiltrating tumors without contrast enhancement, it is also difficult to distinguish between tumor tissue and normal brain. Separating treatment-induced changes from real tumor progression is also challenging, especially if patients have undergone proton radiotherapy [34]. Thus, in the recently updated RANO 2.0 criteria, the postradiotherapy MRI is recommended to be used as the baseline in clinically stable patients [35]. However, treatment-induced image findings may progress for months or years after radiotherapy.

The strength of the present study is the use of real-world individual data from a large cohort of IDH-mutant diffuse supratentorial WHO grade 2 gliomas from three different centers, making the findings more generalizable. Still, despite the relatively long follow-up and a large cohort, conclusions drawn from our assessments may be vulnerable due to the few deaths and censoring, especially for calculations of PPS and OS. Also, MRI examinations included in our observational data were performed with irregular intervals as part of differing clinical routines across multiple centers, and thus there was no central review of this data. Ideally, a validation study for PFS should have access to trial data with both standardized MRI assessments, modern molecular tissue classification, repeated assessments of HRQoL, and very long follow-ups. However, considering the fact that molecular data is incomplete and only 55% of patients reached OS in the RTOG 9802 trial [22], despite nearly 12 years of follow-up, this is currently not realistic.

## Conclusion

In this multicenter cohort study, we observed only a weak correlation between PFS and OS in patients with diffuse supratentorial IDH-mutant WHO grade 2 gliomas. The correlation was especially weak for oligodendrogliomas. Further, a longer PFS was not a predictor of longer PPS. Astrocytoma tumor subtype was the only observed shared predictor for shorter PFS, PPS, and OS. Although PFS is a much-used surrogate endpoint in studies of patients with WHO grade 2 gliomas, it remains unvalidated and demonstrated effects on PFS may not necessarily result in longer PPS or OS.

## Supplementary Material

Progression-free survival versus post-progression survival and overall survival in WHO grade 2 gliomas

## Data Availability

Aggregated data supporting the findings of this study can be made available upon reasonable request. Individual patient data are not available for sharing.
