# Supplementary material for: Progression-free survival versus post-progression survival and overall survival in WHO grade 2 gliomas
Source: Acta Oncol. 2024 Oct 20;63:40845. doi: 10.2340/1651-226X.2024.40845 (PMC11500610; doi:10.2340/1651-226X.2024.40845)
Supplement: Progression-free survival versus post-progression survival and overall survival in WHO grade 2 gliomas [file AO-63-40845-s1.pdf]

Supplementary material has been published as submitted. It has not been copyedited, or typeset by Acta Oncologica

Supplementary table 1. Reoperations during follow up

		Hospital A (n=65)	Hospital B (n=70)	Hospital C (n=184)
Number of reoperations (n, %)				
	No reoperation	22 (34 %)	48 (69 %)	94 (51 %)
	1 reoperation	28 (43 %)	16 (23 %)	70* (38 %)
	≥ 2 reoperations	15 (23 %)	5 (7 %)	19* (10 %)
Time of first reoperation, n (%)				
	Before tumor progression	8 (12 %)	4 (6 %)	17 (9 %)
	At the same day as tumor progression	0 (0 %)	1 (1 %)	19 (10 %)
	≤ 6 months after tumor progression	20 (31 %)	13 (19 %)	41 (22 %)
	6 months to 2 years after tumor progression	13 (20 %)	2 (3 %)	6 (3 %)
	≥ 2 years after tumor progression	2 (3.1 %)	1 (1 %)	7 (4 %)

* Missing data in one patient

Supplementary table 2. Oncological treatments during follow up

		Hospital A (n=65)	Hospital B (n=69*)	Hospital C (n=184)
No radio- or chemotherapy		17 (26 %)	11 (16 %)	35 (19 %)
Both radio- and chemotherapy, n (%)		43 (66 %)	54 (77 %)	107 (58 %)
Radiotherapy only, n (%)		4 (6 %)	2 (3 %)	14 (8 %)
Chemotherapy only, n (%)		1 (2 %)	2 (3 %)	28 (15 %)
Time of first radiotherapy, n (%)				
	≤ 6 months after first surgery	14 (22 %)	26 (37 %)	38 (21 %)
	≥ 6 months after first surgery, before tumor progression	2 (3 %)	11 (16 %)	15 (8 %)
	≤ 6 months after tumor progression	9 (14 %)	11 (16 %)	46 (25 %)
	6 months to 2 years after tumor progression	10 (15 %)	6 (9 %)	14 (8 %)
	≥ 2 years after tumor progression	12 (19 %)	2 (3 %)	8 (4 %)
Time of first chemotherapy, n (%)				
	≤ 6 months after first surgery	10 (15 %)	30 (43 %)	51 (28 %)
	≥ 6 months after first surgery, before tumor progression	1 (2 %)	8 (11 %)	21 (11 %)
	≤ 6 months after tumor progression	7 (11 %)	12 (17 %)	40 (22 %)
	6 months to 2 years after tumor progression	7 (11 %)	3 (4 %)	17 (9 %)
	≥ 2 years after tumor progression	19 (29%)	3 (4 %)	6 (3 %)

* Missing data in one patient
